# Antiepileptic Drug Interactions - Principles and Clinical Implications

**DOI:** 10.2174/157015910792246254

**Published:** 2010-09

**Authors:** Svein I Johannessen, Cecilie Johannessen Landmark

**Affiliations:** 1 The National Center for Epilepsy, Sandvika, and Department of Pharmacology, Oslo University Hospital, Oslo, Norway; 2 Department of Pharmacy, Faculty of Health Sciences, Oslo University College, Oslo, Norway, Postal address: Pilestredet 50, N-0167 Oslo, Norway

**Keywords:** Antiepileptic drugs, interactions, pharmacokinetics, metabolism.

## Abstract

Antiepileptic drugs (AEDs) are widely used as long-term adjunctive therapy or as monotherapy in epilepsy and other indications and consist of a group of drugs that are highly susceptible to drug interactions. The purpose of the present review is to focus upon clinically relevant interactions where AEDs are involved and especially on pharmacokinetic interactions. The older AEDs are susceptible to cause induction (carbamazepine, phenobarbital, phenytoin, primidone) or inhibition (valproic acid), resulting in a decrease or increase, respectively, in the serum concentration of other AEDs, as well as other drug classes (anticoagulants, oral contraceptives, antidepressants, antipsychotics, antimicrobal drugs, antineoplastic drugs, and immunosupressants). Conversely, the serum concentrations of AEDs may be increased by enzyme inhibitors among antidepressants and antipsychotics, antimicrobal drugs (as macrolides or isoniazid) and decreased by other mechanisms as induction, reduced absorption or excretion (as oral contraceptives, cimetidine, probenicid and antacides). Pharmacokinetic interactions involving newer AEDs include the enzyme inhibitors felbamate, rufinamide, and stiripentol and the inducers oxcarbazepine and topiramate. Lamotrigine is affected by these drugs, older AEDs and other drug classes as oral contraceptives. Individual AED interactions may be divided into three levels depending on the clinical consequences of alterations in serum concentrations. This approach may point to interactions of specific importance, although it should be implemented with caution, as it is not meant to oversimplify fact matters. Level 1 involves serious clinical consequences, and the combination should be avoided. Level 2 usually implies cautiousness and possible dosage adjustments, as the combination may not be possible to avoid. Level 3 refers to interactions where dosage adjustments are usually not necessary. Updated knowledge regarding drug interactions is important to predict the potential for harmful or lacking effects involving AEDs.

## INTRODUCTION

Antiepileptic drugs (AEDs) are widely used as long-term adjunctive therapy or as monotherapy in epilepsy and other indications and consist of a group of drugs that are highly susceptible to interactions. During the last years several new AEDs have been marketed. Initially, all new AEDs are licensed for add-on therapy for epilepsy patients. Several AEDs as lamotrigine, valproic acid, oxcarbazepine, carbamazepine, pregabalin, gabapentin, and topiramate are also increasingly used in other indications as psychiatry, neuropathic pain and migraine [1, 2]. Population-based studies of drug utilization demonstrate that 19-24 % of patients with epilepsy use polytherapy with AEDs [2-4]. In recent studies of children and adults with refractory epilepsy, 64 % used polytherapy with two or more AEDs, and 35 % of the adults suffered from CNS-related comorbid conditions, resulting in a considerable risk of interactions [5, 6]. Polytherapy and the potential for interactions with other drugs increase with increasing age, and the elderly is the largest group with newonset epilepsy having a considerable risk of interactions with commonly prescribed drugs [7].

The interactions with older AEDs are thoroughly described in earlier reviews [8-12]. The newer second- and third generation AEDs is less interacting than the older drugs, which results in less complicated therapeutic outcomes and complications for the patients [13]. But, however, since the newer AEDs also often are metabolized in the liver, many of them may cause drug interactions or their serum concentrations be increased or to a lesser extent decreased by the addition of comedication [13-16]. Clearly, the risk of clinically important drug-drug interactions is great in patients with epilepsy, with or without comorbid conditions.

The purpose of the present review is to focus upon clinically relevant interactions between AEDs and AEDs in combination with other important therapeutic drug classes, with emphasis on pharmacokinetic interactions. The first part of the review deals with the principles for pharmacokinetic interactions, including cytochrome P450 (CYP) and uridine glucuronyl transferases (UGT)-mediated enzyme induction and inhibition. In the following sections the implementation of the individual AED interactions in the clinical setting and the consequences of alterations in serum concentrations will be focused upon. This review comprises recent advances regarding drug interactions including new AEDs that have not been described in previous reviews.

## MATERIAL AND METHODS

The present review is based on published articles and searches in PubMed and Google Scholar from July 2008 to May 2010, in addition to references from the included articles. Peer-reviewed articles in English, from international journals, from the earliest relevant data, 1977 to 2010 were included. Primary sources and review articles of importance for the field were used. Published abstracts were included when a complete published article was not available. Unpublished material, single case reports and preclinical studies were not included, but a few exceptions were made where clinical evidence was not available. Negative findings were not included. The searches included combinations of the terms from group 1, 2 and 3:

### Group 1:

CYP, enzyme induction, enzyme inhibition, interaction, metabolism, pharmacology, pharmacokinetics, pharmacodynamics and UGT. 

### Group 2:

Antiepileptic drugs, carbamazepine, clobazam, clonazepam, eslicarbazepine acetate, felbamate, gabapentin, lacosamide, lamotrigine, levetiracetam, oxcarbazepine, phenobarbital, phenytoin, pregabalin, primidone, rufinamide, stiripentol, tiagabine, topiramate, valproic acid, vigabatrin, and zonisamide.

### Group 3:

Antibiotics, antidepressants, antineoplastic drugs, antipsychotics, immunosuppressants, oral coagulants/warfarin, oral contraceptives, psychotropic drugs, other drug classes.

## PHARMACOKINETIC INTERACTIONS

In general, pharmacokinetic interactions may alter absorption, protein binding, metabolism, and excretion of any drug, and these have been investigated in detail for many drugs. They are usually related to alterations in metabolism by enzyme inducers or inhibitors and are often well described in preclinical models. Most drug interactions in the past were discovered due to unexpected change in the clinical status of a patient after addition or withdrawal of a drug from existing medication.

Enzyme induction involves the synthesis of new enzyme, requires protein synthesis and may take many days before it is completed, resulting in increased metabolism, decreased serum concentrations and pharmacological effect (if no active metabolites are present) of the affected drug, and possibly loss of seizure control. The process is reversed when the inducer is withdrawn, resulting in increased serum concentrations and potential for toxic side effects of the affected drug.

Enzyme inhibition results from competition between drugs for the same active site on the enzyme and results in decreased metabolism of the affected drug. Circulating concentrations of the inhibited drug increase to a new steady-state about five half-lives after the interaction. Consequently, pharmacological potentiation will occur quickly if the drug has a short half-life and more slowly if it has a long half-life [12]. Conversely, if the inhibitor is withdrawn, drug concentrations will decrease with risk of seizures. If the drug is a substrate, *in vivo* and *in vitro* inhibition is enzyme-specific and substrate-independent. All drugs that are metabolized to a significant degree by the same enzyme are inhibited by inhibitors of that enzyme and therefore exhibit the same spectrum of interactions. For a given drug the knowledge of the isoform(s) that catalyze(s) its metabolism is important. If the drug is an inhibitor, the potential for any drug to inhibit the various CYPs can be assessed *in vitro* using a specific substrate for those isoforms. If a new drug inhibits one isoform at therapeutic concentrations, it can be predicted that it will interact with any substrate of that isoform [9, 17-19].

### CYP Enzymes and Drug Interactions

There are a number of individual CYP isoenzymes, each of which is a specific gene product with characteristic substrate specificity. The P450 enzyme system consists of a super family of hemoproteins. The nomenclature is based on similarities in amino acid sequences deduced from genes. Each isoform is identified by three terms representing families and subfamilies. An Arabic numeral designates the family (f.ex. CYP2). Isoforms in the same family must have more than 40 % homology in their amino acid sequence. Letters A, B, C, D, and E designate the subfamilies (f.ex. CYP2C). Members of the same subfamily must have more than 55 % homology. The third term, another Arabic numeral, designates a unique gene product with very similar amino acid sequences (f.ex. CYP2C9) [9].

Clinically important CYPs involve certain isoforms that appear to have therapeutic relevance. Only a few of these are important in terms of understanding the interactions of AEDs, CYP1A2, CYP2C9/10, CYP2C19, and CYP3A3/4. Knowledge of the isoenzymes involved in the metabolism of established AEDs allows a prediction of interactions with new drugs in development. Enzyme activity is genetically determined, and pharmacogenetic differences in the metabolic capacity exist for CYP2C9/19 as poor, intermediate and extensive metabolizers, e.g. for CYP2C19 5 % of the Caucasian population is deficient, while 20 % of the Japanese population is deficient [18-20]. CYP2D6 is well described for its pharmacogenetic variability and is involved in the metabolism of commonly used antidepressants and antipsychotics, where 5-10 % of the Caucasian population is deficient, while less than 1 % of the Asian population is deficient. Ultrarapid metabolizers also exist for this enzyme, as more than 20 gene copies may exist in a few percentage of patients [21, 22]. Phenotypically, in clinical practice, pharmacokinetic interactions involving enzyme induction and enzyme inhibition will mimic the genotypes of extensive and poor metabolizers, respectively.

### UGTs and Drug Interactions

The uridine glucuronyl transferases (UGTs) catalyse glucuronidation *via* two enzyme families, UGT1 and UGT2, each with eight isoenzymes identified [10].Glucuronidation is the clearance mechanism of one of ten of the 200 most prescribed drugs in the US [23]. The UGTs are in general less substrate specific, and even though many genetic polymorphisms have been identified, no clear polymodal distribution in genotypes has been identified as for the CYP families. During the last years details in genetics of the UGTs have become available [24, 25]. 

Lamotrigine is metabolized through UGT1A4 [26]. Valproic acid seems to be a substrate for UGT2B7, and polymorphisms exist [18, 27]. As for the CYPs, UGTs are susceptible to induction or inhibition. Probably, their role in the metabolism of AEDs will be closely investigated in the coming years. Pharmacogenetic variability or genetic polymorphisms and variability in the capacity of drug metabolism is an issue that is under investigation [17, 18, 28].

### Pharmacokinetic Interactions and Therapeutic Drug Monitoring (TDM)

	The treatment of epilepsy aims to prevent seizures, and since there is no direct measure to control the pharmacological effect, TDM is an important tool in pharmacovigilance [20]. When a patient is treated with more than one drug, there is often a risk of clinically important drug interactions that may result in altered therapeutic outcome, and interactions are a major contributor to pharmacological variation. TDM may reveal interactions by the measurement of the serum concentrations of AEDs and appropriate dosage adjustments may be necessary [14, 16]. It is important to be observant for loss of efficacy or clinical signs of intoxication and to monitor the drug concentrations closely 2- 4 weeks following addition or withdrawal of a drug. Knowledge of the mechanism of an interaction may allow anticipation of the observed effect.

## PHARMACOKINETIC INTERACTIONS WITH AEDS

Pharmacokinetic interations in the clinical setting may be divided in three levels depending on the magnitude of alterations in serum concentrations and clinical implications. Thus, the most important interactions may be easier to remember (Level 1 and 2 interactions). This approach may point to interactions of specific importance, although it should be implemented with caution, as it is not meant to oversimplify fact matters.

- **Level 1 interactions** may result in potentially serious clinical consequences, and the combination should be avoided
- **Level 2 interactions** usually imply cautiousness and possible dosage adjustments, as the combination may not be possible to avoid
- **Level 3 interactions** refer to interactions where dosage adjustment are usually not necessary, and clinically relevant changes in serum concentrations are not expected

Since several of the older AEDs are well-known enzyme inducers (carbamazepine, phenytoin, phenobarbital, and primidone) or inhibitors (valproic acid), interactions with AEDs are commonly occurring and often have potentially serious clinical implications (Level 1 and 2 interactions). In various instances the knowledge of the possibility of a given interaction may help in better rationalizing the therapeutic approach in avoiding unnecessary risk to the patients. The clinical significance of some of the reported interactions with AEDs may, however, be questioned, if the alterations in serum concentrations are minor (Level 3 interactions). It should also be noted that enzyme-inducing AEDs affect endogenous biochemical pathways, as metabolism of sex hormones, vitamin D homeostasis and bone metabolism and cholesterol synthesis [29, 30].

The newer AEDs are less susceptible to cause pharmacokinetic interactions than the older drugs, but may often be affected by other AEDs or drug classes. Recently, four new AEDs have been marketed (eslicarbazepine acetate, lacosamide, rufinamide, and stiripentol). Lacosamide does not seem to be involved in pharmacokinetic interactions and will not be discussed further [31, 32]. Rufinamide seems to be involved in some interactions, and stiripentol has a greater interaction potential [33-35]. It should, be noted, however, that the use of rufinamide and stiripentol is limited to special pediatric populations.

The main metabolic pathways, enzymes and susceptibility to drug interactions are shown in Table **1**. AEDs with an interaction potential and how they affect other drugs, are shown in Table **2**. Table **3** highlights interactions between other drugs and AEDs. There are more examples listed in Table **2** and **3** than those discussed in the text. Specific combinations and clinical implications of major clinical importance that should be remembered are summarized in Table **4** (Level 1 and 2 interactions).

## INTERACTIONS BETWEEN AEDS

Well-known interactions and newly established interactions of clinical importance will be highlighted. Based on the metabolic pathways described in Table **1**, it is clear that evidence for many possible interactions involving new AEDs is lacking in the literature.

### Enzyme Induction and Possible Loss of Seizure Control

#### Carbamazepine and other Older Enzyme Inducers

Carbamazepine, phenytoin, phenobarbital, and primidone are the major enzyme-inducing AEDs that stimulate the rate of metabolism of most co-administered AEDs, including valproic acid, tiagabine, ethosuximide, lamotrigine, topiramate, oxcarbazepine and its monohydroxy-derivative, zonisamide, felbamate, many benzodiazepines and, to some extent, levetiracetam. This involves various CYP enzymes (CYP1A2, CYP2C9, CYP2C19, CYP3A4), UGTs and epoxide hydrolase. Carbamazepine undergoes autoinduction and also heteroinduction by phenytoin and barbiturates [10, 12]. The active 10,11-epoxide metabolite of carbamazepine is metabolized by epoxide hydrolase. The clinical significance of these interactions is usually modest because the consequences of the reduction in serum concentration of the affected AED are compensated for by the pharmacological effect of the added comedication. However, in some cases, seizure control may be adversely influenced (Level 2 and 3 interactions). Particular caution is required when an enzyme-inducing drug is withdrawn from the therapeutic regimen of patients taking comedications, the metabolism of which has been increased by the inducing drug. In fact, the concentration of these drugs may increase to toxic concentrations after removal of the inducing drug unless their dosage is adjusted appropriately (Level 2 interactions). Dosage adjustments of the AED affected by the interaction are most commonly required for valproic acid, lamotrigine and tiagabine, because the decrease in the serum concentrations of these drugs after adding carbamazepine can be quite prominent (50-75 %) [12,  36]. Furthermore, the central nervous system adverse effects of carbamazepine may be potentiated by lamotrigine and by oxcarbazepine [12, 37]. The effects of carbamazepine on the pharmacokinetics of phenobarbital and primidone are somewhat variable. In patients on primidone, carbamazepine may decrease the serum concentrations of primidone and increase those of metabolically derived phenobarbital. Phenytoin and barbiturates decrease serum carbamazepine concentrations. 

The classical enzyme-inducing AEDs may also increase the metabolism of several new AEDs (Level 2 interactions). The half-life of felbamate is decreased from up to 22 hours to about 14 hours [38, 39]. The metabolism of lamotrigine is increased in combination with enzyme inducers, shortening the half-life from 30 to 15 hours on average [40, 41]. The clearance of levetiracetam has been shown to be approximately 25-37 % higher in patients also treated with enzyme inducing AEDs [42-44]. Interactions between oxcarbazepine and carbamazepine, phenytoin and valproic acid were investigated in a controlled study with 43 patients and showed that oxcarbazepine did not affect any of the drugs, while induction by carbamazepine and phenytoin decreased the AUC of the main metabolite, 10-OH-carbazepine, (MHD) by 40 % and 29 %, respectively [45]. The metabolism of oxcarbazepine (600 mg single dose) was induced by about 30 % by phenobarbital, as seen in the lower plasma concentration of MHD, while no change was seen with valproic acid [46]. A recent study suggests that enzyme-inducing agents may cause a 20- 30 % reduction in the plasma concentration of pregabalin at steady state [47]. Even though pregabalin is metabolized only to a minor extent, it is supposed that patients on long-term enzyme-inducing drugs might metabolize pregabalin somewhat more extensively than expected from healthy volunteers [47]. Enzyme-inducing AEDs increase the clearance of tiagabine by 50-65 % and subsequently, reduce the half-life on average from 7 hours to 2-5 hours [48]. These drugs also reduce the serum concentrations of topiramate by about 50 %, as measured in 94 children and adults [49]. The half-life of zonisamide is reduced by 25-35 % by inducing AEDs (phenytoin and carbamazepine) [50]. Comedication with enzyme inducing AEDs decreased plasma concentrations of rufinamide by up to 46 %, which may be explained by induction of carboxylesterases [12]. Patients treated with enzyme inducing AEDs exhibit higher clearance of stiripentol to a variable extent [51]. 

#### Newer AEDs

Oxcarbazepine may act as a weak enzyme inducer or inhibitor, but enzyme induction is probably the most clinically relevant mechanism (Table **1**). It induces the UGT-mediated metabolism of lamotrigine, as seen by a 29 % decrease in the serum concentration [40, 52]. Felbamate may act as a weak inducer of CYP3A4 and decreases the serum concentrations of carbamazepine by 25 % and increases the concentrations of carbamazepine-10,11-epoxide [53]. Felbamate also increases the formation of the active metabolite of clobazam, n-desmethyl-clobazam several-fold [54]. Findings from pharmacokinetic modeling indicate that rufinamide may slightly increase the metabolism of carbamazepine and lamotrigine (less than 20 %) [35]. Enzyme induction by newer AEDs is clearly less prominent than by older AEDs, and they may be regarded as Level 2-3 interactions.

### Enzyme Inhibition by AEDs with Risk of Intoxication

#### Valproic Acid

Valproic acid is a broad enzyme inhibitor, including CYPs, UGTs and epoxide hydrolase, and inhibits the metabolism of lamotrigine (glucuronidation), phenobarbital (oxidation), the metabolite 10,11-epoxide of carbamazepine (epoxide hydrolase), and ethosuximide (oxidation) leading to increased serum concentrations of the inhibited drugs and consequently, an increased risk of toxicity [9, 55]. The serum concentration of lamotrigine is increased by 211 % by valproic acid, as studied retrospectively in patients, increasing the half-life from 30 to 60 hours [41]. This is of great importance because the risk of skin rashes induced by lamotrigine is dependent on the rate of increase of the serum concentration of lamotrigine. The addition of lamotrigine to existing valproic acid treatment, therefore calls for a low starting dose and cautious dose escalation (Level 2 interaction). Lamotrigine may also affect the metabolism of valproic acid, as shown in a study where the addition of lamotrigine to valproic acid therapy led to a 25 % increase in valproic acid serum concentrations [56]. Serum phenobarbital concentrations may increase considerably after adding valproic acid [36]. A dose reduction of phenobarbital by up to 80 % may be necessary to avoid side effects [55]. If valproic acid is added to carbamazepine, neurotoxic signs may develop due to an increase in the serum concentration of its epoxide metabolite caused by inhibition of the epoxide hydrolase [12]. Valproic acid increases rufinamide concentrations by about 70 %, especially in children compared to patients without valproic acid as comedication, and this finding may be explained by inhibition of carboxylesterases [35]. In adults, the estimated reduction in rufinamide clearance by valproic acid was 17 % [34].

#### Felbamate

Felbamate is a potent and broad ranging inhibitor, including CYP2C19 and may increase plasma concentrations of phenobarbital, phenytoin and valproic acid significantly [39,57-60]. The clinical importance of felbamate is, however, limited because of the diminished use of the drug during the last years due to toxic effects. 

#### Oxcarbazepine

Oxcarbazepine is a weak inhibitor of CYP2C19 and may increase the serum concentration of phenytoin and phenobarbital [61].

#### Rufinamide

The new AED rufinamide has demonstrated to moderately increase serum concentrations of carbamazepine, phenobarbital and phenytoin (6-17 %), possibly due to enzyme inhibition [35]. The clinical importance of these interactions is uncertain.

#### Stiripentol

Stiripentol is extensively metabolized through four main pathways, and it exhibits non-linear kinetics [62] (Table **1**). This drug is a potent inhibitor of CYP3A4, 1A2 and 2C19 and increases the serum concentration of other AEDs as phenytoin, carbamazepine, phenobarbital, valproic acid, and clobazam [63-65] (Level 2 interactions). Consequently, the concentrations of their metabolites are decreased, which may result in increased tolerability of the original drugs, as for carbamazepine and its epoxide metabolite [64].

Even if the clinical use of felbamate, rufinamide, and stiripentol is limited, potential interactions are important to be aware of.

## INTERACTIONS BETWEEN AEDS AND OTHER DRUGS

Interactions between AEDs and other drugs are described in detailed reviews [7, 11, 19, 66, 67]. They may result in alterations in serum concentrations of the actual AED(s) or the other drug(s), often caused by induction or inhibition of CYP enzymes. In the following sections, commonly occurring interactions will be elucidated. In general, the newer AEDs are less suceptible for drug interactions than the older ones. Interactions where AEDs are affecting other drugs are shown in Table **2**, while interactions where other drugs are affecting AEDs (antidepressants, antipsychotics, antimicrobal drugs, and others) are shown in Table **3**. Clearly, interactions between new AEDs and other drugs are scarce in the literature, and in many cases, oral contraceprives (OCs) and warfarin are best documented. Some negative findings regarding other therapeutic classes have, however, been published. No interactions between OCs and gabapentin, levetiracetam, pregabalin, tiagabine, vigabatrin and zonisamide have been reported [68-73].

### Enzyme Induction or Inhibition by AEDs

For female patients, possible interactions between AEDs and OCs are of major importance, as a large portion (>50 %) of patients lack knowledge and information [74, 75]. The older enzyme-inducing AEDs and the newer, oxcarbazepine, lamotrigine, felbamate, topiramate (>200 mg), eslicarbazepine acetate, and rufinamide stimulate the metabolism of OCs involving CYP3A4. Women who use AEDs that interact with hormonal contraceptives (estrogen or gestagen) should be advised to use non-hormonal contraceptive methods [76] (Level 1 interactions).

#### Carbamazepine and other Older Enzyme-Inducing AEDs

Carbamazepine induces the metabolism of various CYPs and UGTs and thus many other drugs (including oral anticoagulants, ciclosporin A, and many antineoplastic agents) and may have important clinical implications and possible therapeutic failure of the affected drug (Level 1 and 2 interactions). Carbamazepine (600 mg/day) increases the clearance of ethinyl estradiol and norethindrone by 127 % and 69 %, respectively [77]. Regarding psychotropic drugs, carbamazepine reduces the serum concentrations of both the older typical and newer atypical drugs, including risperidone, clozapine, olanzapine, quetiapine, ziprasidone, aripiprazol, haloperidol, chlorpromazine and older antidepressants as clomipramine and imipramine [66, 67, 78] (Level 2 interactions). The interaction is greatest with drugs that undergo significant first-pass metabolism, such as itraconazole, praziquantel, indinavir. Most dihydropyridine calcium antagonists are also affected [7]. Addition of simvastatin to healthy volunteers taking carbamazepine lead to a 75-82 % decrease in the AUC of simvastatin treated compared to controls, possibly by induction of CYP 3A4 [79]. In enzyme-induced patients the serum concentration of these drugs may decrease 5-10-fold, and the practical management of these patients may be very difficult. In case of warfarin treatment, it is important to be aware of the potential danger if the inducing drug is discontinued, where there is a risk of haemorrhage (Level 2 interaction).

The other older AEDs with enzyme-inducing properties also affect antipsychotic drugs (Level 2 interactions). Phenobarbital  decreases the serum concentration of clozapine, haloperidol and chlorpromazine, and phenytoin decreases the serum concentration of quetiapine, clozapine, haloperidol and chlorpromazine [66]. Phenobarbital and phenytoin also decrease serum concentrations of older antidepressants as clomipramine and imipramine [7].

#### Eslicarbazepine Acetate

Eslicarbazepine acetate (1200 mg daily) reduces the effectiveness of OCs, by a decrease of the AUC of levonorgestrel and ethinyloestradiol by 37% and 42%, respectively, due to enzyme induction [80] (Level 1 interaction, according to recent guidelines [76]).

#### Felbamate

In the limited cases where felbamate is used, interactions with OCs and warfarin must be expected. In a randomized controlled study with female volunteers, felbamate decreased the AUC of gestodone by 42 %, but not ethinyl estradiol, and therefore, the use of OCs with a low-dose of estrogen is not advised [81] (Level 1 interaction). In patients treated with warfarin and felbamate, a dose reduction of warfarin was necessary to maintain its anticoagulant efficacy [82] (Level 2 interaction). 

#### Oxcarbazepine

Oxcarbazepine induces the metabolism of OCs, ethinyl estradiol and levonorgestrel, as their AUCs were reduced by 47 %, accompanied by a 45 % decrease in their half-lives, by concomitant administration of oxcarbazepine (maintenance dose 1200 mg/day) as studied in healthy women [83] (Level 1 interactions).

#### Topiramate

Topiramate used in daily doses of 50-200 mg/day does not significantly affect serum concentrations of OCs containing ethinyl estradiol and norethindrone [84]. In higher doses (up to 400 mg/day), however, a modest inducing effect was seen with a 18-33 % increase in oral clearance [85] (Level 1 interaction). Topiramate in combination with hydrochlortiazide, metformin or pioglitazone may require dosage adjustments of either drug and should be monitored closely, and carboanhydrase inhibitors should be avoided [86] (Level 2-1 interaction).

#### Rufinamide

Rufinamide increases the clearance of OCs caused by weak enzyme induction, as shown by a reduction in plasma concentrations of ethinyl estradiol and norethindrone of 22 % and 14 %, respectively [35, 87]. Rufinamide increased the clearance of triazolam by 55 % in a study with healthy volunteers, possibly by enzyme induction [35]. 

#### Stiripentol

Due to the inhibitory effect of stiripentol on several CYPs, the dose of concomitantly used drugs should be reduced by 50 % if they are eliminated through the CYP P450 system, including drugs used for anesthesia, hypertension, diabetes, and asthma. Warfarin should be avoided [88]. However, further studies are needed.

#### Valproic Acid

The potent enzyme inhibitor valproic acid has the potential to increase the serum concentrations and risk of toxicity of many other drugs, and some of them are documented (Level 2 interactions). Valproic acid inhibits the metabolism of amitriptyline and nortriptyline and increases their total AUC by 42 %, as studied in healthy subjects [89]. There is conflicting evidence whether valproic acid affects antipsychotics, as described for the enzyme inducers [37, 90]. Valproic acid treatment results in a three-fold increase in the incidence of haematological adverse effects associated with antineoplastic drugs as cisplatin and etoposide caused by enzyme inhibition [91].

### Enzyme Induction or Inhibition by Other Drugs

#### Carbamazepine

Many drugs other than AEDs affect carbamazepine metabolism. Several drugs, including known inhibitors of CYP3A4, can precipitate signs of carbamazepine toxicity by increasing serum carbamazepine concentrations. For example, the antibiotic agents clarithromycin, troleandomycin and erythromycin, and the analgesic drugs dextropropoxyphene increase serum carbamazepine concentrations markedly and should be avoided in patients taking carbamazepine [92-94] (Level 1 interactions). Probenecid appears to reduce the serum concentration of carbamazepine by increasing its biotransformation to carbamazepine-10,11-epoxide [95]. Of the antipsychotic drugs, risperidone, and possibly haloperidol are the only drugs that have been shown to increase the carbamazepine serum concentration [37]. 

Grapefruit juice may also inhibit CYP3A4, and has a modest elevating effect on serum carbamazepine concentrations. St. John’s Wort has the potential to increase the metabolism of AEDs, since it induces CYP3A4, CYP2C9 and CYP2C19, possibly by affecting drug transporter activity in the gastrointestinal tract [23, 96, 97]. Interactions between AEDs and herbal medicines are not sufficiently investigated in clinical studies, but there is a potential for pharmacokinetic interactions [98, 99].

#### Phenobarbital and Phenytoin

Phenobarbital and phenytoin follow the same metabolizing pathways as carbamazepine, and most of the drugs that affect carbamazepine are also expected to affect these drugs, as for instance the antidepressant clomipramine or the antibiotic isoniazid [11, 12, 37, 67].

#### Lamotrigine

Recently, interactions between lamotrigine and OCs have been closely investigated. In a study with 22 women using lamotrigine (on average 350 mg/day) and OCs and 30 women on lamotrigine (on average 350 mg/day) without OCs, lamotrigine serum concentrations were reduced by more than 50 %, from 28 to 13 µmol/L [100, 101]. Furthermore, Reimers *et al*. [102] found that it is ethinyl estradiol and not progesterone that reduces the lamotrigine concentrations. This interaction is likely to be caused by stimulation of UGT1A4 activity by the steroids and may result in reduced seizure control in some women. The serum concentrations of lamotrigine should therefore be closely monitored, and this interaction is of major importance, as lamotrigine is often a preferred choice of drug in female patients (Level 1 interaction, according to [76]). Rifampicin has been shown to increase lamotrigine clearance of about 50 % [103].

#### Oxcarbazepine

It is possible that oxcarbazepine is affected in a similar way due to a common metabolic pathway [76] (Level 1 interactions). 

#### Valproic Acid

Valproic acid is affected by OCs by a similar mechanism as lamotrigine, but less pronounced, as demonstrated by an increase of 22 % and 45 % increase in the apparent clearance of total and unbound valproic acid, respectively [104]. The serum concentrations of valproic acid should be closely monitored [76] (Level 1-2 interaction). Valproic acid clearance is decreased moderately by chlorpromazine (15 %) [105]. Carbapenem antibiotics as imipenem, meropenem and panipenem also decrease serum levels of valproic acid, by enzyme inhibition and potentially other mechanisms [106].

## OTHER MECHANISMS

### Absorption

Gabapentin has shown great variability in absorption from the gastrointestinal tract, and the absorption may be reduced by up to 24 % with some antacids, as well as its renal clearance may be reduced by cimetidin [107, 108]. The intake of rufinamide with food increases the C_max_ by >50 % and AUC with 30-40 % [35].

### Protein Binding

Phenytoin, valproic acid, and tiagabine are highly bound to serum proteins, and displacement from protein binding sites may occur, especially the displacement of phenytoin by valproic acid [12]. In addition, stiripentol is 99 % protein bound [62], and displacement interactions are therefore also likely to occur, but studies are lacking. Usually, these interactions are not clinically important (Level 3 interactions), but may be of importance for the interpretation of TDM data. The total concentration of the affected drug is decreased, but the concentration of the unbound, pharmacologically active drug is not altered. Valproic acid displaces tiagabine from its binding sites at serum proteins concentration-dependently [109]. Since tiagabine is present in nanomolar concentrations in the blood, it is not expected to displace compounds with therapeutic concentrations in the micromolar range, as valproic acid or phenytoin [10]. Other highly protein-bound drugs, as salicylates and naproxen, displace tiagabine from serum proteins and slightly decrease the total serum concentration [109].

### P-glycoprotein and Related Transporters

P-glycoprotein is a part of the superfamily consisting of various transporters or efflux pumps called ATP-binding cassette (ABC) involved in multidrug resistance (MDR) and is an expanding area in pharmacogenetics [27, 110]. Drugs that induce or inhibit CYP enzymes may also affect the expression of P-glycoprotein or other transporters involved in MDR in the gastrointestinal tract, kidneys and other tissues and are important mechanism involved in drug resistance in epilepsy [111]. Overexpression of these transporters has been observed in the brain of patients with resistant epilepsy, and the overexpression of P-glycoprotein in excretory organs suggests that it has a central role in drug elimination and may be coupled to subtherapeutic serum concentrations of AEDs [112]. Recently, carbamazepine, phenytoin, primidone, valproic acid, and lamotrigine were explored in an *in vitro* model for three ABC transporters including P-glycoprotein (ABC(B1)), but none of the drugs demonstrated to modulate the transporter activity directly [110]. Another study, however, demonstrated that phenytoin and phenobarbital were substrates of human P-glycoprotein, and directional transport was determined for lamotrigine and levetiracetam, but not for carbamazepine [113]. A recent study including cohorts from various ethnic groups of patients with epilepsy demonstrated that there were no correlation between a specific polymorphism (3435C>T) in the ABCB1 gene and response to AED treatment one year after the first seizure [114]. The possible role of P-glycoprotein and related transporters regarding pharmacokinetic interactions and pharmacogenetic variability is poorly investigated for AEDs in humans and needs more attention.

### Pharmacodynamic Interactions

Pharmacodynamic interactions are interactions at the site of action of the drugs and may involve synergistic or antagonistic alteration, and they are not possible to measure and evaluate, as no alterations in pharmacokinetics and consequently, serum concentrations are observed. Pharmacodynamic interactions with most CNS-active drugs may affect efficacy and tolerability, but they are difficult to evaluate in controlled studies. These interactions are most often not intended, are poorly investigated mechanistically and are often based on emipirical observations. They should, however, be considered for the individual patient. In preclinical models, possible synergistic effects between newly developed AEDs are investigated, but clinical evidence is lacking as these interactions are rarely described or documented. A synergistic pharmacodynamic interaction between lamotrigine and valproic acid has, however, been demonstrated in an open cross-over study with 20 adult patients with refractory complex partial seizures The dose of both drugs, however, may need to be reduced to minimize the risk of intolerable side effects [115]. Preclinical studies have suggested a supra-additive or synergistic pharmacodynamic effect by e.g. combining levetiracetam with carbamazepine, felbamate, oxcarbazepine or topiramate, and similarly with gabapentin and vigabatrin, as demonstrated in the maximal electroshock-induced seizure model in mice [116-119]. On the other hand, lamotrigine in combination with carbamazepine or oxcarbazepine resulted in an antagonistic effect [116]. Other combinations of AEDs may give rise to excessive adverse reactions, which may be explained as a pharmacodynamic interaction, as lamotrigine and carbamazepine or oxcarbazepine or levetiracetam and topiramate [12, 37] The use of psychotropic drugs in patients with epilepsy is common, and they may affect seizure threshold and contribute to CNS-related adverse events [78]. 

## PERSPECTIVES AND CONCLUDING REMARKS

- Newer AEDs have a less potential for pharmacokinetic interactions than older AEDs, but they are susceptible to interactions, since they are often used as adjunctive therapy with older AEDs. The older AEDs may cause interactions involving enzyme induction or inhibition, affecting new AEDs as well as other drugs like anticoagulants, OCs, other CNS-active drugs, immunosuppressants, antimicrobal drugs as isoniazid or macrolides. Based upon comparative studies and evidence-based guidelines, newer AEDs are considered to be as efficacious as the older drugs and better tolerated. One has to keep in mind that the older enzyme-inducing AEDs affect endogenous biochemical pathways as well as a variety of drugs and therefore, newer and non-inducing AEDs may be preferable when initiating AED therapy [29].
- Documented interactions involving new AEDs are as yet limited. Among the newer AEDs, lamotrigine is one of the most commonly used, and its metabolism is reduced when added to valproic acid, increased when added to older enzyme-inducing AEDs, or increased by adding OCs. Other newer AEDs for which data on pharmacokinetic interactions have been documented include felbamate, oxcarbazepine, topiramate, rufinamide, and stiripentol.
- Reliable drug surveillance systems for adverse drug reactions often caused by drug interactions are important to detect and follow interactions as closely as possible. These should involve health care professionals and the patients, in national or interational reporting systems, as the WHO Drug Monitoring Programme [120, 121].
- Several new AEDs are undergoing late-stage clinical trials, including brivaracetam, carisbamate and retigabine [122, 123]. Their interaction potential compared to existing drugs will be further investigated in patients during the next few years.
- Polytherapy may be a rational strategy in the treatment of many patients, and studies designed to evaluate specific AED combinations should be conducted [124]. The implementation of drug interactions in the clinical setting with focus on each AED is important to predict the consequences of alterations in serum concentrations. By the categorization of pharmacokinetic interactions from Level 1-3, their clinical importance may be more clearly evaluated and easier to remember. Updated knowledge regarding drug interactions is important to predict the potential for harmful or lacking effects of AEDs.

## Figures and Tables

**Table 1:** AEDs and their Main Mechanisms of Elimination and Susceptibility to Pharmacokinetic Interactions

AED	Main Route of Elimination	CYP Degradation	CYP Induction	CYP Induction	UGT Degradation	UGT Induction	UGT Inhibition
Carbamazepine	Oxidation	Yes, 3A4, and epoxide hydrolase (metabolite)	Yes, CYP3A4, 2C9, 1A2	No	No	Yes	No
Clobazam	Oxidation	Yes, CYP3A4	No	No	No	No	No
Clonazepam	Oxidation	Yes, CYP3A4	No	No	No	No	No
Eslicarbazepine acetate	Glucuronidation	No	Yes, CYP3A4	No	Yes, but isoenzymes not identified	No	No
Ethosuximide	Oxidation	Yes, CYP3A4	No	No	No	No	No
Felbamate	Oxidations (>50 %), renal excretion (<30 %)	Yes, CYP 3A4, 2E1	CYP3A4*	CYP2C19	No	No	No
Gabapentin	Renal excretion	No	No	No	No	No	No
Lacosamide	Demethylation	No	No	No	No	No	No
Lamotrigine	Conjugation	No	No	No	Yes, UGT1A4	No	No
Levetiracetam	Hydrolysis (25 %), renal excretion (75 %)	No, type-B esterase	No	No	No	?	No
Oxcarbazepine	Conjugation (>50 %), renal excretion (>30 %)	No, arylketone reductase	Yes, CYP3A4,	Yes, CYP2C19*	Yes	Yes, UGT1A4	No
Phenobarbital	Oxidation/conjugation (75 %), renal excretion (25 %)	Yes, CYP2C9, 2C19, 2E1	Yes, CYP3A4, 2C9, 1A2	No	Yes	No	No
Phenytoin	Oxidation	Yes, CYP2C9, 2C19	Yes, CYP3A4,2C9, 1A2	Yes, CYP2C9	No	Yes	No
Pregabalin	Renal excretion	No	No	No	No	No	No
Rufinamide	Hydrolysis, glucuronidation	No, carboxyl esterases	Yes, CYP3A4	No	Yes	No	No
Stiripentol	Oxidation, hydroxylation,O-methylation, glucuronidation	No, carboxyl esterases	No	Yes, CYP 1A2, 3A4, 2C19, 2D6	No	No	No?
Tiagabine	Oxidation	Yes, CYP3A4	No	No	No	No	No
Topiramate	Oxidation (20-60 %), renal excretion (40-80 %)	Yes, but isoenzymes not identified	Yes, CYP3A4* (>200 mg/day)	Yes, CYP2C19*	No	No	No
Topiramate	Oxidation (>50 %), conjugation (30-40 (30-40 %)	Yes, 2A6, 2C9, 2C19, 2B6 and mitochondrial oxidases	No	Yes, CYP2C9, CYP3A4?, and epoxide hydrolase	Yes, UGT1A3, 2B7	No	Yes
Vigabatrin	Renal excretion	No	No	No	No	No	No
Zonisamide	Oxidation, reduction, acetylation (>50 %), renal excretion (30 %)	Yes, CYP3A4, and N-acetyl transferase	No	No	No	No	No

* Weak induction or inhibition. AED=Antiepileptic drug. CYP=Cytochrome P450 enzyme, UGT=Uridine diphosphate glucuronosyltransferase enzymes.

The most commonly used AEDs are listed. Main routes of metabolism and affection of other enzymes are listed. Isoenzymes are given where they have been identified. Several sources are used, see text.

**Table 2:** Clinically Important Interactions Between AEDs and with other Drug Classes

Affected Drug Classes					
AEDs Susceptible to Interactions	AEDs	Antidepressants and Antipsychotics	Oral Contraceptives	Antimicrobal Drugs	Various (e.g. Warfarin, Antineoplastic Drugs, Immuno-Suppressants)
*Enzyme inducers that will decrease serum concentrations of affected drugs*					
Carbamazepine, phenobarbital, phenytoin, primidone	Benzodiazepines, ethosuximide, lamotrigine, oxcarbazepine, pregabalin, rufinamide, stiripentol, tiagabine, topiramate, zonisamide, valproic acid,	*Typical:* Chlorpromazine, haloperidol *Atypical:* Aripiprazol, clozapine, olanzapine, quetiapine, risperidone, ziprasidone Antidepressants: Clomipramine Imipramine	Estrogen component of combination pills	Doxycycline, indinavir, itraconazole, metronidazol, praziquantel	Warfarin Antineoplastic agents (e.g. cyclophosphamide, irinotecan, methotrexate, tamoxifen) Immuno-suppressants: Ciclosporin, tacrolimus Varia: Cortisol derivatives, dextropropoxyphene, dihydropyridine calcium antagonists, fentanyl, statines, methadone, theophylline, thyroxine
Eslicarbazepine and oxcarbazepine	Lamotrigine, phenobarbital, phenytoin, (mainly induction)		Estrogen component of combination pills		
Felbamate	Carbamazepine Clobazam				
Topiramate	Phenytoin (in some cases)		Estrogen component of combination pills (topiramate doses >200 mg/day)		Carboanhydrase inhibitors, digoxin, hydrochlortiazide, metformin, pioglitazone,
*Enzyme inhibitors that will increase serum concentrations of affected drugs*					
Valproic acid	Carbamazepine, ethosuximide, lamotrigine, phenobarbital, rufinamide	Amitriptyline, nortriptyline		Carbapenem antibiotics: Imipenem, meropenem, panipenem	Cisplatin,etoposide
Felbamate	Clonazepam phenobarbital, phenytoin, valproic acid		Estrogen component of combination pills		Warfarin
Rufinamide	Carbamazepine, clobazam, phenytoin, phenobarbital, valproic acid		Estrogen component of combination pills		Triazolam
Stiripentol	Carbamazepine, clobazam, phenytoin, phenobarbital, valproic acid				Various potential interactions*

AEDs=Antiepileptic drugs. The list is not all-including but relevant examples are given. Several references are used, see text for details and selected reviews, [7-13] and the spc of the various drugs. Oral contraceptives and warfarin are described in more detail in Table 4. ^*^ *In vitro* studies suggest a potential for interactions with most drug classes metabolized by CYP3A4, 1A2, 2C19.

* *In vitro* studies suggest a potential for interactions with most drug classes metabolized by CYP3A4, 1A2, 2C19.

**Table 3:** Other Drugs Affecting Commonly used AEDs. Examples from Therapeutic Drug Classes of Clinical Importance

Therapeutic Drug Classes	Affected AEDs	Mechanism of Interaction and Clinical Consequence

**Antidepressants and antipsychotics**		Enzyme inhibition leading to increased serum concentrations of AEDs
Haloperidol, risperidone	Carbamazepine	
Chlorpromazine	Valproic acid	
Clomipramine	Carbamazepine, phenytoin, phenobarbital, valproic acid	
Sertraline	Carbamazepine, lamotrigine, phenytoin, valproic acid,	

**Oral contraceptives**	Lamotrigine, valproic acid (oxcarbazepine?)	Induction of metabolism (glucuronidation) and reduced serum concentrations of AEDs

**Antimicrobal drugs**		Enzyme inhibition by antimicrobal drugs leading to increased serum concentrations of AEDs
Macrolides (clarithromycin, erythromycin, troleandomycin)	Carbamazepine	
Rifampicin	Lamotrigine	
Isoniazid	Carbamazepine, ethosuximide, phenytoin, valproic acid	

**Others**		

Probenicid	Carbamazepine	Induction of metabolism and reduced serum concentrations of carbamazepine

	
Antacides	Gabapentin	Decreased absorption of gabapentin
Cimetidine		Reduction in excretion of gabapentin leading to a prolonged half-life

Salicylates and naproxene	Tiagabine	Displacement of tiagabine from plasma proteins leading to a decrease in the total serum concentration of tiagabine but unchanged free concentration
	

AEDs=Antiepileptic drugs. The list is not all-including, but relevant examples are given. Several references are used, see text for details and selected reviews [7-13] and the spc of the various drugs.

**Table 4:** Clinically Important Drug Combinations Involving AEDs (Level 1-2)

AED	Added Drug	Clinical Consequence	Level of Importance (1-2)	Precautions
Carbamazepine	Oral contraceptives	Induction of estrogen metabolism, reduction in serum concentrations, and loss of contraceptive effect	Level 1: Should be avoided	Avoid the combination (or use of oral contraceptives with >50 µg ethinylestradiol), utilize barrier contraception. Addition of 4 mg folic acid daily for women of child bearing potential if used
Carbamazepine	Antibiotics: Clarithromycin, erythromycin, troleandomycin	Inhibition of carbamazepine metabolism, elevated serum concentrations, giving rise to potential serious toxicity if the antibiotics are added	Level 1: Should be avoided	Avoid macrolide antibiotics that inhibit CYP3A4, prefer azithromycin or spiramycin
Carbamazepine	Dextropropoxyphene	Inhibition of carbamazepine metabolism, elevated serum concentrations, giving rise to potential serious toxicity if the analgesic drug is added	Level 1: Should be avoided	The combination should be avoided.
Lamotrigine	Oral contraceptives	Induction of lamotrigine metabolism, reduction in serum concentrations by 50 %, and reduced seizure control, if OCs are added	Level 1: Should be avoided	The combination should be avoided. Alternatively, increase in lamotrigine dose and monitor closely
Valproic acid	Lamotrigine	Inhibition of lamotrigine metabolism and elevated serum concentrations giving rise to skin rashes, or neurotoxic effects if lamotrigine is added to valproic acid A synergistic pharmacological effect and improved seizure control	Level 2: Dosage adjustments and monitoring are needed	Low initial dose and slow titration of lamotrigine dose when initiating therapy, about 50 % of the dose used in monotherapy is required A dose reduction of both drugs may reduce risk of adverse effects without affecting the efficacy
Valproic acid	Phenobarbital	Inhibition of phenobarbital metabolism resulting in elevated serum concentrations, and risk of intoxication if valproic acid is added as a second drug	Level 2: Dosage adjustments and monitoring are needed	A reduction in phenobarbital dose by up to 80 %
Carbamazepine (or phenobarbital, phenytoin, primidone)	Oral anticoagulant: Warfarin	Induction of warfarin metabolism, reduced serum concentrations, increasing the risk of coagulation that may be fatal if enzyme-inducing AEDs are added	Level 2: Dosage adjustments and monitoring are needed	An increase in the warfarin dose required to maintain the INR, close monitoring of INR.
Carbamazepine (or phenobarbital, phenytoin, primidone)	Immunsuppressants: Ciclosporin, tacrolimus	Induction of immunosuppressant metabolism, reduction in serum concentrations, and potential therapeutic failure if enzymeinducing AEDs are added	Level 2: Dosage adjustments and monitoring are needed	Increase in the dose of immunosuppressant to avoid therapeutic failure, important for drugs with a narrow therapeutic range

AEDs=Antiepileptic drugs. Several references are used, see text for details.
